# ^1^H, ^15^N and ^13^C chemical shift assignments of the La motif and RRM1 from human LARP6

**DOI:** 10.1007/s12104-015-9605-3

**Published:** 2015-04-22

**Authors:** Luigi Martino, Nicholas J. H. Salisbury, Paul Brown, Geoff Kelly, R. Andrew Atkinson, Maria R. Conte

**Affiliations:** Randall Division of Cell and Molecular Biophysics, King’s College London, New Hunt’s House, Guy’s Campus, London, SE1 1UL UK; MRC Biomedical NMR Centre, MRC National Institute for Medical Research, The Ridgeway, Mill Hill, London, NW7 1AA UK; Division of Molecular Structure, MRC National Institute for Medical Research, The Ridgeway, Mill Hill, London, NW7 1AA UK; Department of Biochemistry, University of Cambridge, Cambridge, CB2 1QW UK

**Keywords:** LARP6, La motif, RRM1, La related protein, RNA binding protein

## Abstract

We report here the nearly complete ^1^H, ^15^N and ^13^C resonance assignment of the La motif and RNA recognition motif 1 of human LARP6, an RNA binding protein involved in regulating collagen synthesis.

## Biological context

LARP6 is a member of the La related proteins (LARP) superfamily and it has been implicated in several developmental events including myogenesis, neurogenesis and possibly metastasis (Bayfield et al. 2010; Bousquet-Antonelli and Deragon 2009). In vertebrates, LARP6 regulates collagen synthesis by binding to a conserved stem-loop in the 5′ untranslated region (UTR) of the mRNAs encoding the collagen α1(I) and α2(I) subunits, thereby coordinating their translation into the heterotrimeric collagen type I (Blackstock et al. 2014; Cai et al. 2010a, b). This interaction is mediated by a conserved RNA binding unit present in LARP6, named the La module, which comprises two domains, a La motif (LaM) and an RNA recognition motif (RRM1). The La module was first discovered in the founding member of the LARPs, the La protein, where the LaM and RRM1 were shown to work in synergy to recognise 3′ UUU_OH_ RNA targets (Alfano et al. 2004; Bayfield et al. 2010; Kotik-Kogan et al. 2008). Although the La module is conserved across the LARP superfamily, the recognised RNA targets are not, and this RNA binding versatility is thought, at least in part, to account for the different cellular processes in which LARPs are involved (Bayfield et al. 2010). Contrary to the archetype La protein, for which high resolution structures of several domains in the apo and bound form, as well as biophysical insights into its RNA binding properties, have been reported (Jacks et al. 2003; Alfano et al. 2004; Teplova et al. 2006; Kotik-Kogan et al. 2008; Martino et al. 2012), the LARPs are much less well understood and the mechanism by which La modules of LARPs can recognise a great variety of RNA molecules, with different shapes and sequences, is still elusive.

To understand in detail the RNA recognition mechanism of LARPs we embarked on a structural and biophysical analysis of the LaM and RRM1 from human LARP6 (HsLARP6). Interestingly, this study revealed that the relative orientation of the LaM and RRM1, mainly dictated by the sequence and structure of the interdomain linker, could play a key role in RNA target discrimination by the La module (Martino et al. 2015). These investigations illustrate the complexity of protein-RNA recognition by underscoring the importance of modular types of interaction in achieving binding specificity and affinity.

Since malfunction of collagen production is connected to a number of fibroproliferative disorders the investigation of the RNA binding mechanism of human LARP6 may be exploited in the future for rational drug design. Here we report the backbone and the sidechain NMR assignment of the LaM and RRM1 of human LARP6.

## Protein expression and purification

For our NMR studies, two human LARP6 domains, the LaM, encompassing residues 70–183, and RRM1, residues 180–295, were prepared as follows. Both domains were cloned into pET-Duet1 vector (Novagen) with an N-terminal histidine tag followed by a TEV-cleavage site. ^15^N and ^15^N/^13^C labelled recombinant proteins were produced in *Escherichia coli* Rosetta II, growing transformed bacteria in minimal media enriched with 0.8 g L^−1^^15^N-ammonium chloride and 2 g L^−1^^13^C glucose, and induced at 18 °C for 14 h. Cell pellets were resuspended in 50 mM Tris, pH 8.0, 300 mM NaCl, 10 mM imidazole, 5 % glycerol, 2 mM PMSF (phenylmethanesulfonyl fluoride) and lysozyme, and lysed by sonication. Following centrifugation, the proteins were purified by nickel affinity chromatography on a 5 mL HisTrap column (GE Healthcare) following the manufacturer’s protocol. The N-terminal histidine tag was removed by overnight incubation with TEV protease (TEV^pro^) (at TEV^pro^:HsLARP6 molar ratio of 1:50) at 4 °C in 50 mM Tris, pH 8.0, 100 mM KCl, 0.2 mM EDTA, 1 mM dithiothreitol (DTT). The reaction mixture was subsequently applied to a Ni–NTA column (Qiagen) to remove the cleaved tags, the His_6_-tagged TEV^pro^ and any undigested product, and the cleaved LARP6 proteins were dialysed overnight in 50 mM Tris pH 7.25, 100 mM KCl, 0.2 mM EDTA, 1 mM DTT. The proteins were finally purified on a 5 mL Hi-Trap heparin column (GE Healthcare) and eluted with a linear 0–2 M KCl gradient in 25 mM Tris pH 7.25, 10 % glycerol, 1 mM DTT. The LaM and RRM1 were dialysed in 20 mM Tris pH 7.25, 100 mM KCl, 50 mM arginine glutamate salt (Golovanov et al. 2004), 1 mM DTT and 20 mM Tris pH 7.25, 100 mM KCl, 1 mM DTT respectively.

## NMR spectroscopy

NMR samples contained ~0.5 mM protein in 95 % H_2_O/5 % D_2_O or 99 % D_2_O at pH 7.25 in 100 mM KCl, 50 mM arginine glutamate salt, 1 mM DTT for the LaM and in 20 mM Tris pH 7.25, 100 mM KCl, 1 mM DTT for RRM1. All NMR spectra for backbone and sidechain resonance assignment were collected at 298 K on a Varian Inova spectrometer operating 18.8 T and on Bruker Avance spectrometers at 14.1 and 16.4 T equipped with triple resonance cryoprobes. Backbone resonances were assigned in a sequential manner using [^1^H,^15^N]-HSQC, HNCA, HN(CO)CA, HNCACB, HN(CO)CACB and HNCO experiments (Grzesiek and Bax 1993). Sidechain resonances were obtained using [^1^H,^15^N]-HSQC, [^1^H,^13^C]-HSQC HCCH-TOCSY^15^N-edited NOESY-HSQC and ^13^C-edited NOESY-HSQC spectra (Fesik et al. 1988). NMR data were processed using NMRPipe/NMRDraw (Delaglio et al. 1995) and visualised/assigned using CcpNMR Analysis 2.2 (Vranken et al. 2005) software and/or CARA/Neasy software (Bartels et al. 1995). Chemical shifts were referenced to internal 4,4-dimethyl-4-silapentane-1-sulfonic acid (DSS).

## Extent of the assignment and data deposition

**Fig. 1:**
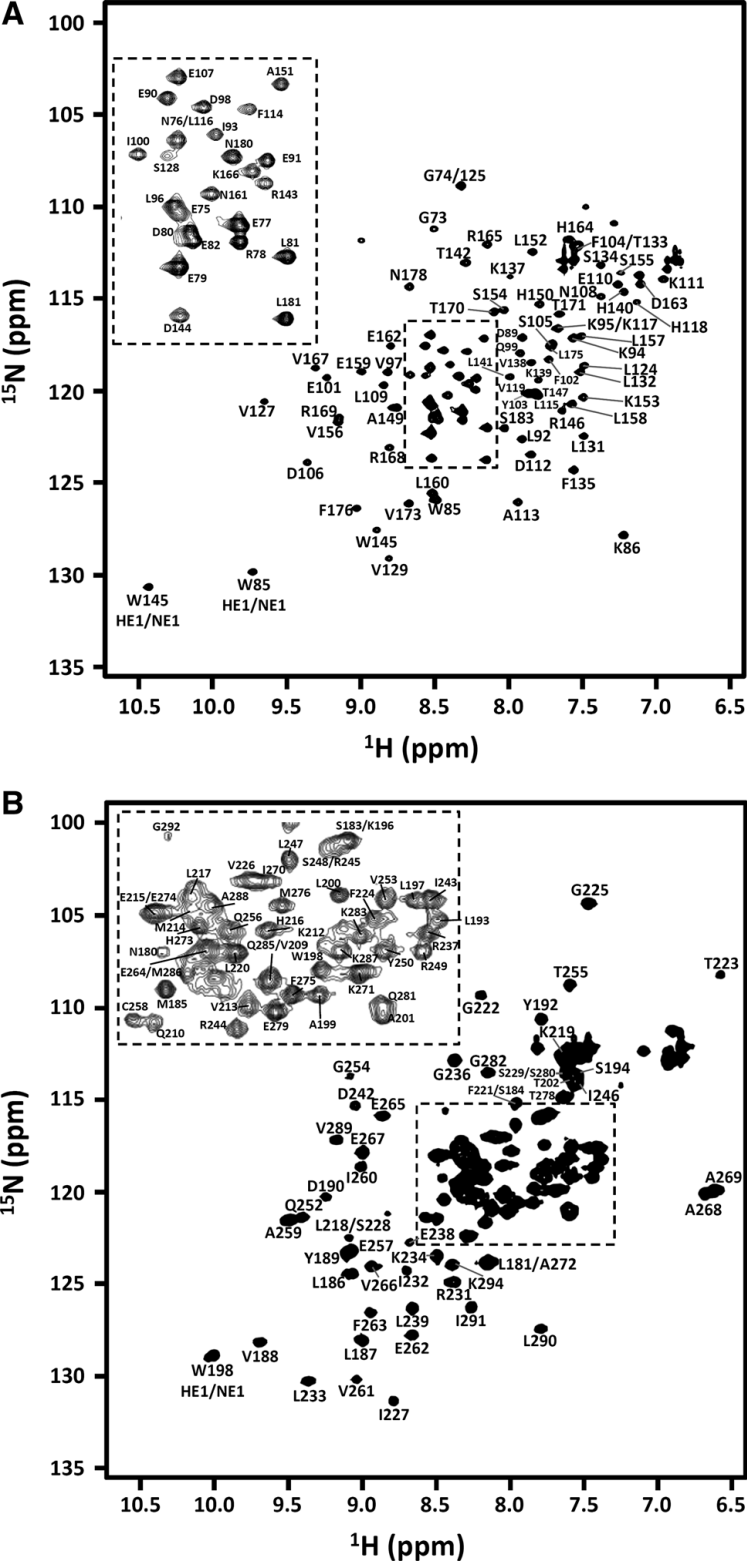
[^1^H, ^15^N]-HSQC spectra for human LARP6 LaM (**a**) and RRM1 (**b**) recorded at 298 K on a Bruker Avance spectrometer working at 16.4 T. Backbone chemical shifts assignments are labelled for both domains. *Residue types* and *numbers* are indicated. Residue numbering corresponds to native sequence. For both spectra, top *left panels* in *dotted squares* show an expansion of the central crowded [^1^H, ^15^N] HSQC region for clarity. The difference in linewidths between the two domains reflects a different sample behavior in the experimental conditions used, confirmed by relaxation analysis (Martino et al. 2015)

Figure 1 shows the assigned [^1^H, ^15^N]-HSQC spectra for the LaM (a) and RRM1 (b) acquired at 298 K. The dispersion of the resonances in both cases suggests that both domains are well folded. The LaM is made of 114 residues of which 6 are prolines and 3 are glycines. For the backbone, 97/107 NH, 102/114 Hα, 84/114 CO, 106/114 Cα and 100/111 Cβ resonances were successfully assigned. This corresponds to 90, 89, 74, 92 and 90 % of the NH, Hα, CO, Cα and Cβ resonances respectively and implies an 87 % complete backbone assignment. Around 78 % of aliphatic sidechain (position γ onwards) and 65 % of the aromatic side chains ^1^H and ^13^C assignments have also been made. The RRM1 has 116 residues with 7 prolines and 7 glycines. Here the backbone assignment was 88 % complete: assignments have been obtained for 104/108 NH (95 %), 97/116 Hα (84 %), 93/116 CO (80 %), 111/116 Cα (96 %) and 93/109 Cβ (85 %) resonances. In addition, around 82 % and 72 % of aliphatic and aromatic ^13^C and ^1^H sidechain resonances have been assigned respectively.

Most of the missing assignments in the two domains correspond to residues T70, A71, S72, Q83, R120, R121, N122, K123, Y126, K130, K136 for the LaM and Q204, K205, N206, G207, S251 for the RRM1, for which peaks in the [^1^H,^15^N]-HSQC spectra could not be observed. None of the exchangeable sidechain protons of Arg and Lys residues was identified, nor the sidechain amide groups of most Asn and Gln.

Secondary structures were derived from backbone chemical shifts and estimates for ψ/φ dihedral angles were obtained using TALOS+ (Shen et al. 2009). As expected, the secondary structure elements predicted for HsLARP6 LaM closely resemble what was previously found for human La LaM (Alfano et al. 2003). Interestingly, the topology for the HsLARP6 RRM1 was found to be β1α0′α1β2α1′β3α2β4; this domain therefore contains two non-canonical secondary structure elements located in the loop between β1 and α1 (α0′) and the loop between β2 and β3 (α1′) in addition to the expected four β-strands (β1–β4) and two α-helices (α1 and α2) typical of the RRM fold. These findings were confirmed in the high resolution structure recently obtained (Martino et al. 2015).

The chemical shift data were deposited in the BioMagResBank (http://www.bmrb.wisc.edu/) under the accession numbers 25159 and 25160 for the LaM and RRM1 respectively.
